# Laparoscopic surgery for locally advanced T4 colon cancer: the long-term outcomes and prognostic factors

**DOI:** 10.1007/s00595-017-1621-8

**Published:** 2017-12-29

**Authors:** Takahiro Yamanashi, Takatoshi Nakamura, Takeo Sato, Masanori Naito, Hirohisa Miura, Atsuko Tsutsui, Masashi Shimazu, Masahiko Watanabe

**Affiliations:** Department of Surgery, Kitasato University School of Medicine, 1-15-1 Kitasato, Minami-ku, Sagamihara, Kanagawa 252-0374 Japan

**Keywords:** Colorectal cancer, T4 colon cancer, Laparoscopic surgery, Long-term outcomes, Prognostic factors

## Abstract

**Purpose:**

For locally advanced pathological T4 (pT4) colon cancer, the safety and feasibility of laparoscopic procedures remain controversial. Therefore, this study aimed to assess short-term and long-term outcomes and to identify the prognostic factors in laparoscopic surgery for pT4 colon cancer.

**Methods:**

The study group included 130 patients who underwent laparoscopic radical resection for pT4 colon and rectosigmoid cancer from January 2004 through December 2012. The short-term outcomes, long-term outcomes, and prognostic factors in pT4 colon cancer were analyzed.

**Results:**

The median operative time was 205 min, with a median blood loss of 10 ml. The conversion rate was 3.8%, and 13 patients (10.0%) had postoperative complications. The radial resection margin was positive in 1 patient (0.8%). The median follow-up time was 73 months. The 5-year overall survival (OS) and recurrence-free survival (RFS) were 77.2 and 63.5%, respectively. On a multivariate analysis, a male sex [hazard ratio (HR) 3.09, *p* < 0.001], lymph node ratio ≥ 0.06 (HR 2.35, *p* = 0.021), tumor diameter < 38 mm (HR 2.57, *p* = 0.007), and right-sided colon cancer (HR 2.11, *p* = 0.047) were significantly related to a poor OS.

**Conclusions:**

These results suggest that laparoscopic surgery for pT4 colon cancer is safe and feasible, and the oncological outcomes are acceptable. Based on the present findings, select patients with locally advanced colon cancer should not be excluded from laparoscopic surgery.

## Introduction

Laparoscopic surgery for advanced colorectal cancer has become widespread, with demonstrated short-term benefits and better long-term oncological outcomes than open surgery [1–10]. However, for locally advanced pathological T4 (pT4) colon cancer based on the American Joint Committee on Cancer (AJCC) TNM staging system [11], the safety and feasibility of laparoscopic procedures remain controversial. In pT4 colon cancer, technically demanding surgical procedures, including en bloc resection of adjacent infiltrated organs or structures, are generally required. It is well known that open multivisceral resection for pT4 colon cancer has a high postoperative morbidity and a high risk of microscopically positive surgical margins [12, 13]. For these reasons, some authors consider pT4 colon cancer to be a relative contraindication to laparoscopic surgery that may result in prolonged operative time, an increased conversion rate, higher postoperative morbidity, and, most importantly, suboptimal oncological results [6].

There is a general lack of evidence-based literature concerning laparoscopic surgery for locally advanced colon cancer. Indeed, cases of clinical T4 (cT4) tumor, perforated tumor, and acute bowel obstruction were excluded in large randomized, controlled trials comparing the laparoscopic approach with the open approach for colorectal cancer [1–7]. Some retrospective studies have reported satisfactory surgical and oncological outcomes of laparoscopic surgery for T4 colon cancer [14–22], but these studies were relatively small series, or the duration of follow-up was short in some reports, and the prognostic factors of patients with pT4 colon cancer who underwent laparoscopic surgery were not reported.

Therefore, this study aimed to retrospectively assess the short-term and long-term outcomes and identify prognostic factors of laparoscopic resection for pT4 colon cancer, in a large series of 130 patients with a long median follow-up period of 73 months.

## Methods

### Patients

Laparoscopic surgery for advanced colorectal cancer has been performed in our hospital since 1995. This retrospective study was approved by the local ethics board. The study included 130 patients who underwent radical laparoscopic resection for pT4 colon and rectosigmoid (RS) cancer without transverse colon cancer, descending colon cancer, or RS cancer requiring low anterior resection, excluding those with distant metastases, from January 2004 through December 2012 at our institution. Patients with severe medical conditions or with definite contiguous organ involvement on preoperative imaging, including cT4b tumor, were also excluded.

All patients underwent a preoperative evaluation including a physical examination, colonoscopy with a biopsy, and chest-abdominopelvic computed tomography (CT) with contrast enhancement. In suspicious cases, positron emission tomography (PET) was performed to identify distant organ metastases. Preoperative laboratory data included a complete blood cell count, biochemical profile, and tumor marker [carcinoembryonic antigen (CEA)] levels. Patients with non-metastatic colon cancer did not receive neoadjuvant chemotherapy. Colonic obstruction was managed using preoperative endoscopic stent insertion followed by laparoscopic resection.

### Operative procedures and follow-up

Patients underwent standard preoperative preparation. All laparoscopic surgeries were radical resections with a complete mesocolic excision and central vascular ligation by colorectal surgeons with extensive experience. Laparoscopic procedures were performed using a pure laparoscopic technique. The appropriate surgical procedures were selected based on tumor location as follows: ileocecal resection (ICR), right hemicolectomy (RHC), sigmoidectomy (SR), and anterior resection (AR). Extracorporeal anastomosis was performed for right-sided colon cancer. The extraction incision was shielded using a wound protector before the specimen was removed. Multivisceral en bloc resection with a 1-cm margin of normal tissue was performed for tumor with involvement to adjacent organs or structures.

After recovery from surgery, adjuvant chemotherapy by oncologists was recommended for patients with TNM stage III disease or those with unfavorable histopathological characteristics, unless there were contraindications related to a patient’s performance status. All patients were followed regularly using an oncological follow-up program for at least 5 years after surgery or until death in cases of recurrence. Patients were followed-up every 3 months for the first 3 years and every 6 months thereafter. Blood test results, including CEA, were checked at each visit. Chest-abdominopelvic CT was performed annually. Colonoscopy was performed 1 year after surgery and every 2 years thereafter. If recurrence was suspected, magnetic resonance imaging (MRI) and/or PET-CT was used to confirm the diagnosis of metastases. Biopsies were selectively performed. Operation was considered for patients with good performance status with resectable recurrence. The follow-up time was calculated as the time interval from the operation until death and not until the last follow-up date.

### Study outcomes

Patient age, sex, body mass index (BMI), American Society of Anesthesiologists (ASA) score, CEA level, clinical TNM staging, tumor location, operative procedure, anastomosis, operative time, blood loss, conversion to open surgery, number of harvested lymph nodes, lymph node ratio (LNR), histopathological TNM staging, lymphatic invasion, vascular invasion, tumor differentiation, tumor maximum diameter, radial resection margin, first flatus, duration of hospital stay, postoperative morbidity, mortality, adjuvant chemotherapy, and long-term oncological outcomes were obtained from the medical records. Conversion from laparoscopy to open surgery was defined as whenever the specimen extraction wound was enlarged for a reason other than delivery of the specimen. The ratio of the number of metastatic lymph nodes to the total number of harvested lymph nodes (LNR) has shown prognostic significance in colorectal cancer [23–26]. A receiver operating characteristic (ROC) curve analysis identified 0.06 as the best LNR cut-off value that had an impact on the overall survival (OS) in the present study, with an area under the curve (AUC) of 0.68. For the analysis of the prognosis, the cut-off value of each clinicopathological factor was determined using an ROC curve analysis as follows: age, 74 years; BMI, 21 kg/m^2^; CEA, 4.8 ng/ml; number of harvested lymph nodes, 17; maximum tumor diameter, 38 mm; operative time, 230 min; and blood loss, 35 ml. pN2 patients (≥ 4 positive nodes) have been shown to have a poorer prognosis in stage III based on the AJCC TNM staging system than pN1 patients (≤ 3 positive nodes) [27]. In the present study, pN was subdivided into pN0–1 and pN2. In addition, cases with vascular invasion have also shown a poor prognosis in colorectal cancer. A previous report showed that the degree of vascular invasion influences liver metastasis, local recurrence, and survival rates [28]. In the present study, lymphatic invasion and vascular invasion were divided into 4 grades: ly/v0, which included no evidence of vessel permeation by tumor cells; ly/v1, which included the possible or doubtful presence of vessel permeation; ly/v2, which included the definite presence of permeation to a few vessels; and ly/v3, which included the definite presence of permeation to many vessels. Furthermore, vascular invasion (or lymphatic invasion) was subdivided into v0–2 (or ly0–2) and v3 (or ly3) for the analysis of the prognosis.

Postoperative morbidity and mortality were defined as events occurring during the hospital stay or within 30 days after surgery. Postoperative complications were categorized by the Clavien–Dindo classification [29]. All such events were assessed by a clinician and documented prospectively in the database. The long-term oncological outcomes included recurrence, the OS, and the recurrence-free survival (RFS). Prognostic factors for the OS and RFS in pT4 colon cancer were analyzed using the clinicopathological parameters and perioperative outcomes.

### Statistical analyses

Statistical analyses were performed using statistical software JMP pro 11 (SAS Institute Inc., Cary, NC, USA). Descriptive data are presented as the mean and standard deviation (SD), median and range, and number of patients and percentage. A significant difference was defined as a *p* value < 0.05. The OS and RFS at 5 years were analyzed using the Kaplan–Meier method and compared between subgroups using the log-rank test. Prognostic factors for the OS and RFS were analyzed by Cox regression hazard models, including all variables reaching *p* values < 0.1 on the univariate analyses.

## Results

All 130 patients with pT4 colon and RS cancer who underwent radical laparoscopic resection between January 2004 and December 2012 were selected for study analyses with a median follow-up period of 73 months (range 12–162). The clinicopathological and perioperative findings for all 130 patients are summarized in Tables 1 and 2, respectively. The mean age was 64.2 (± 11.5) years. There were 73 male patients (56.2%) and 57 female patients (43.8%). The mean CEA level was 9.7 (± 14.0) ng/ml. Tumor locations were the cecum in 25 patients (19.2%), ascending colon in 30 patients (23.1%), sigmoid colon in 63 patients (48.5%), and RS colon in 12 patients (9.2%). Right-sided colon cancer was present in 55 patients (42.3%), with left-sided colon cancer in 75 patients (57.7%). The median operative time was 205 (range 105–460) min, with a median blood loss of 10 (range 5–655) ml. Multivisceral resection was required in 16 patients (12.3%), as follows: abdominal wall in 8 patients (6.2%); retroperitoneal adipose tissue in 3 patients (2.3%); partial resection of the bladder in 2 patients (1.5%); small intestine in 1 patient (0.8%); sigmoid colon in 1 patient (0.8%); and seminal duct in 1 patient (0.8%). Conversion from laparoscopy to open surgery was required in 5 patients (3.8%) due to technical difficulties with multivisceral resection of the abdominal wall in 3 patients, the small intestine in 1 patient, and the sigmoid colon in 1 patient. The median first flatus was 1 day (range 1–8 days). The median postoperative hospital stay was 7.5 (range 4–278) days. Overall, 13 patients (10.0%) had postoperative complications classified as Clavien–Dindo II and III, with anastomotic leakage in 4 patients (3.1%), bowel obstruction in 2 patients (1.5%), and wound infection in 7 patients (5.4%). There were no cases of mortality at 30 days postoperatively. Pathological stage (pStage) was IIB in 44 patients (33.8%), IIC in 3 patients (2.3%), IIIB in 56 patients (43.1%), and IIIC in 27 patients (20.8%). A total of 83 patients (63.8%) had lymph node metastases. The mean number of harvested lymph nodes was 19.9 (± 9.3). In 107 patients (82.3%), more than 12 lymph nodes were harvested. The mean LNR was 0.12 (± 0.16). The mean tumor maximum diameter was 47.3 (± 18.0) mm. The radial resection margin was positive in 1 patient (0.8%); although the *T* factor of this patient was pT4a, it was difficult to maintain the operative field because of the presence of strong tumor-induced inflammatory adhesions. The R0 resection rate was 99.2% (129/130). Ultimately, 67 patients (51.5%) received postoperative adjuvant chemotherapy. The chemotherapy rate for each pStage was as follows: 12.8% in pStage II (6/47 patients); 71.4% in pStage IIIB (40/56 patients); and 77.8% in pStage IIIC (21/27 patients).

**Table 1:** Clinicopathological characteristics of patients with pT4 colon and rectosigmoid cancer (*N* = 130)

Parameters	Categories	Number (%)
Age, mean ± SD, years		64.2 ± 11.5
Gender	Male	73 (56.2)
	Female	57 (43.8)
Body mass index, mean ± SD		22.3 ± 3.1
ASA score^a^	1	56 (43.1)
	2	62 (47.7)
	3	12 (9.2)
CEA, mean ± SD, ng/ml		9.7 ± 14.0
Tumor location (1)	Cecum	25 (19.2)
	Ascending	30 (23.1)
	Sigmoid	63 (48.5)
	Rectosigmoid	12 (9.2)
Tumor location (2)	Right-sided	55 (42.3)
	Left-sided	75 (57.7)
Operative procedure	ICR	20 (15.4)
	RHC	35 (26.9)
	SR	63 (48.5)
	AR	12 (9.2)
Anastomosis	DST	68 (52.3)
	FEEA	62 (47.7)
Depth of invasion (cT)	cT3	32 (24.6)
	cT4a	98 (75.4)
Lymph node metastasis (cN)	cN0	61 (46.9)
	cN1	64 (49.2)
	cN2	5 (3.8)
Distant metastasis (cM)	cM0	130 (100.0)
	cM1	0 (0.0)
Clinical stage (UICC 7th)	IIA	25 (19.2)
	IIB	36 (27.7)
	IIIB	65 (50.0)
	IIIC	4 (3.1)
Depth of invasion (pT)	pT4a	125 (96.2)
	pT4b	5 (3.8)
Lymph node metastasis (pN)	pN0	47 (36.2)
	pN1	56 (43.1)
	pN2	27 (20.8)
Distant metastasis (pM)	pM0	130 (100.0)
	pM1	0 (0.0)
Pathological stage (UICC 7th)	IIB	44 (33.8)
	IIC	3 (2.3)
	IIIB	56 (43.1)
	IIIC	27 (20.8)
Harvested lymph nodes, mean ± SD		19.9 ± 9.3
Lymph node ratio^b^, mean ± SD		0.12 ± 0.16
Tumor differentiation	Well	39 (30.0)
	Moderately	81 (62.3)
	Poorly	10 (7.7)
Lymphatic invasion	ly0	16 (12.3)
	ly1	54 (41.5)
	ly2	45 (34.6)
	ly3	15 (11.5)
Vascular invasion	v0	2 (1.5)
	v1	45 (34.6)
	v2	50 (38.5)
	v3	33 (25.4)
Tumor diameter, mean ± SD, mm		47.3 ± 18.0
Radial resection margin	Negative	129 (99.2)
	Positive	1 (0.8)
Adjuvant chemotherapy	Absence	63 (48.5)
	Presence	67 (51.5)

*ICR* ileocecal resection, *RHC* right hemicolectomy, *SR* sigmoidectomy, *AR* anterior resection, *DST* double stapling technique, *FEEA* functional end-to-end anastomosis, *SD* standard deviation

^a^American Society of Anesthesiologists score

^b^The ratio of metastatic lymph nodes to the total number of harvested lymph nodes

**Table 2:** Perioperative outcomes (*N* = 130)

Parameters	Number (%)
Operative time, median (range), min	205 (105–460)
Blood loss, median (range), ml	10 (5-655)
Conversion to open surgery	5 (3.8)
First flatus, median (range), day	1 (1–8)
Postoperative hospital stay, median (range), day	7.5 (4-278)
Mortality	0 (0.0)
Morbidity (Clavien–Dindo^a^ ≥ Grade II)	13 (10.0)
Anastomotic leakage	4 (3.1)
Bowel obstruction	2 (1.5)
Wound infection	7 (5.4)

^a^Clavien–Dindo classification

**Fig. 1:**
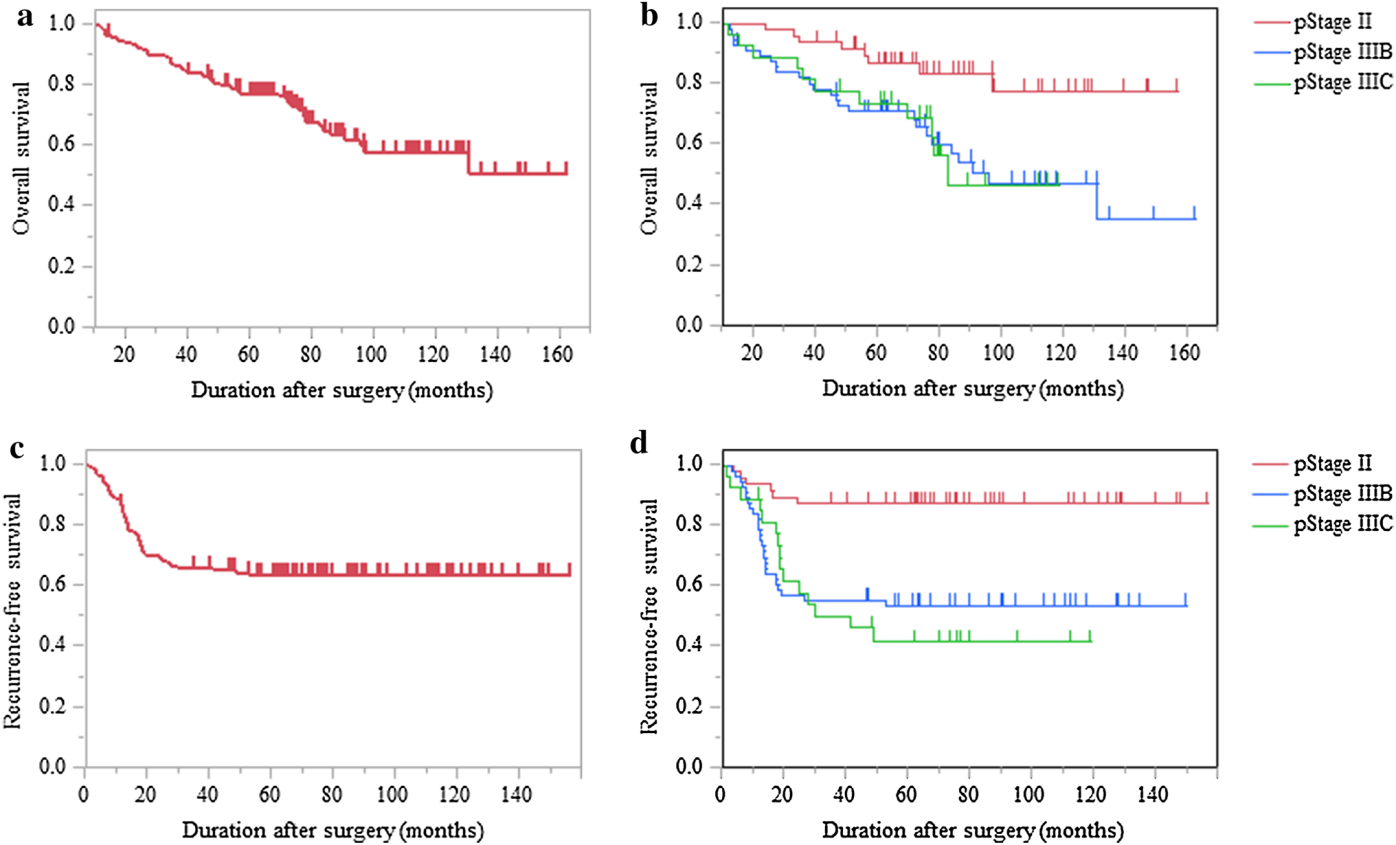
The survival of patients with pT4 colon and rectosigmoid cancer (*N* = 130). **a** The overall survival (OS) in all cases. **b** OS, pStage is subdivided into II, IIIB, and IIIC. **c** The recurrence-free survival (RFS) in all cases. **d** RFS, pStage is subdivided into II, IIIB, and IIIC. Patients with pStage II had a significantly better prognosis than patients with IIIB (*p* = 0.003 for OS, *p* < 0.001 for RFS) and IIIC (*p* = 0.016 for OS, *p* < 0.001 for RFS)

The OS and RFS curves are shown in Fig. 1. The 5-year OS and RFS were 77.2 and 63.5%, respectively. The pStage was subdivided into II, IIIB, and IIIC; the 5-year OS was 86.6% in II, 70.9% in IIIB, and 73.7% in IIIC, and the 5-year RFS was 87.2% in II, 53.5% in IIIB, and 41.7% in IIIC. Patients with pStage II had a significantly better prognosis than patients with IIIB (*p* = 0.003 for OS, *p* < 0.001 for RFS) and IIIC (*p* = 0.016 for OS, *p* < 0.001 for RFS). There were no significant differences in the OS and RFS between pStage IIIB and IIIC. Overall, 47 patients (36.2%) had recurrences, involving the liver in 16 patients (12.3%), peritoneum in 12 patients (9.2%), lung in 11 patients (8.5%), paraaortic region in 8 patients (6.2%), local region in 5 patients (3.8%), metachronous colorectal cancer in 3 patients (2.3%), and ovary in 2 patients (1.5%), including double-counted patients (Table 3).

**Table 3:** Initial recurrence site in pT4 colon and rectosigmoid cancer (*N* = 130)

	Number (%)	Surgical procedure
Recurrence (total)	47/130 (36.2)	
Liver	16 (12.3)	Resection in 5, RFA^a^ in 1
Peritoneum	12 (9.2)	Resection in 1
Lung	11 (8.5)	Resection in 3
Paraaortic region	8 (6.2)	Resection in 1
Local region	5 (3.8)	Resection in 1, enterostomy in 2
Metachronous CRC	3 (2.3)	Resection in 2
Ovary	2 (1.5)	Resection in 1
Stage II	6/47 (12.8)	
Liver	3 (2.3)	Resection in 1
Peritoneum	1 (0.8)	Absence
Lung	2 (1.5)	Absence
Paraaortic region	1 (0.8)	Absence
Local region	1 (0.8)	Enterostomy in 1
Metachronous CRC	0 (0.0)	Absence
Ovary	0 (0.0)	Absence
Stage III	41/83 (49.4)	
Liver	13 (10.0)	Resection in 4, RFA^a^ in 1
Peritoneum	11 (8.5)	Resection in 1
Lung	9 (6.9)	Resection in 3
Paraaortic region	7 (5.4)	Resection in 1
Local region	4 (3.1)	Resection in 1, enterostomy in 1
Metachronous CRC	3 (2.3)	Resection in 2
Ovary	2 (1.5)	Resection in 1

*CRC* colorectal cancer

Including double-count patients

^a^Radio frequency ablation

**Fig. 2:**
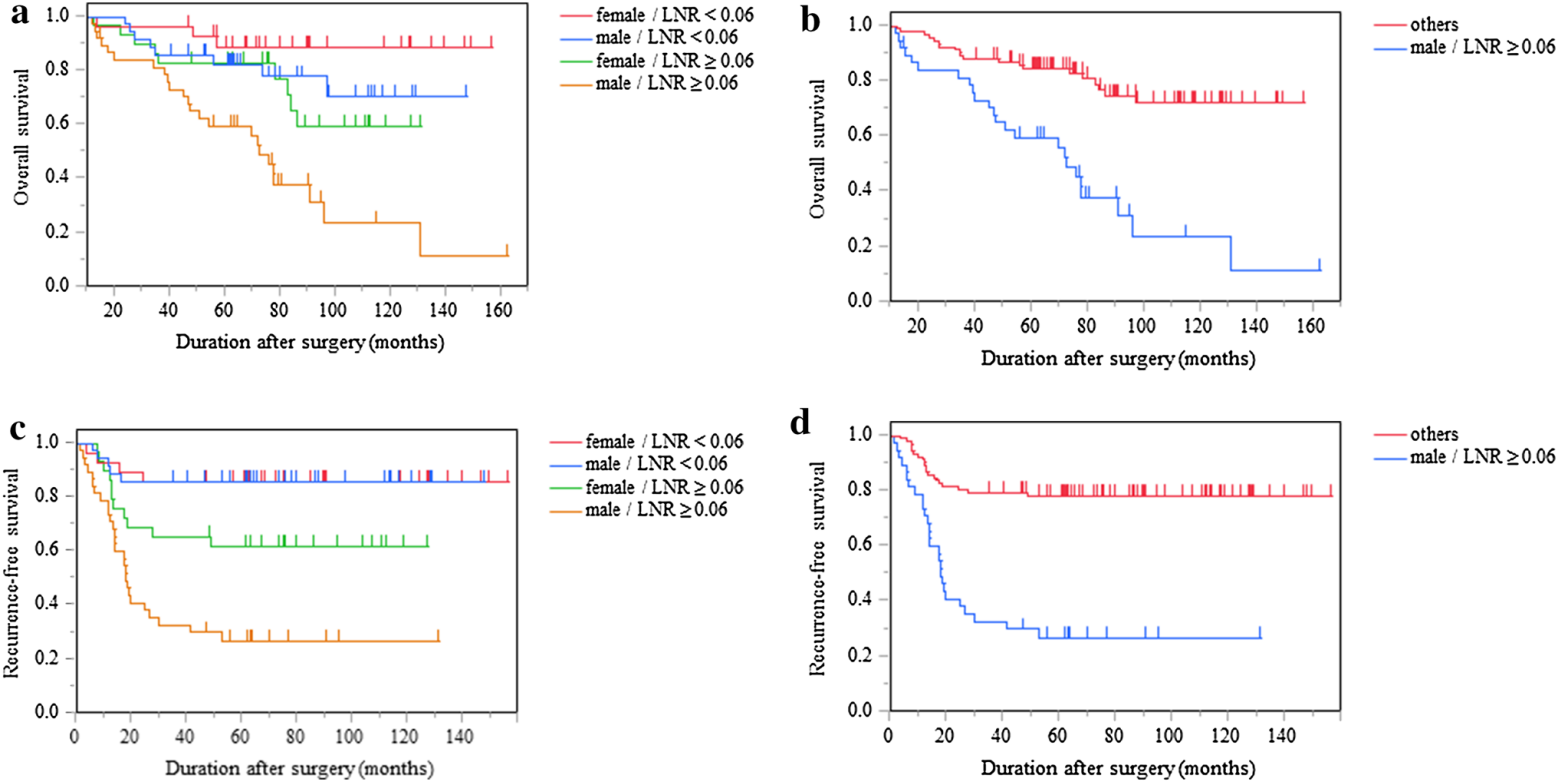
The survival of patients with pT4 colon and rectosigmoid cancer in a subgroup analysis (*N* = 130). **a** OS, all cases are subdivided into 4 groups as follows: female with LNR < 0.06, male with LNR < 0.06, female with LNR ≥ 0.06, and male with LNR ≥ 0.06. **b** OS, all cases are subdivided into 2 groups as follows: male with LNR ≥ 0.06, and others. **c** RFS, all cases are subdivided into 4 groups as follows: female with LNR < 0.06, male with LNR < 0.06, female with LNR ≥ 0.06, and male with LNR ≥ 0.06. **d** RFS, all cases are subdivided into 2 groups as follows: male with LNR ≥ 0.06, and others. *LNR* lymph node ratio, the ratio of the number of metastatic lymph nodes to the total number of harvested lymph nodes. Female patients with LNR < 0.06 had a significantly better OS and RFS than male patients with LNR ≥ 0.06 (both *p* < 0.001). Male patients with LNR ≥ 0.06 had a significantly worse OS and RFS than the others (both *p* < 0.001)

The results of the univariate and multivariate analyses are shown in Tables 4 and 5, respectively. On univariate analyses for the OS, possible prognostic factors, including the age (*p* = 0.003), sex (*p* = 0.003), CEA (*p* = 0.011), tumor location (*p* = 0.068), LNR (*p* < 0.001), lymphatic invasion (*p* = 0.009), vascular invasion (*p* = 0.038), and tumor diameter (*p* = 0.047), were selected for multivariate analyses. For the RFS, the sex (*p* = 0.033), CEA (*p* = 0.070), pN (*p* = 0.045), LNR (*p* < 0.001), tumor differentiation (*p* = 0.097), lymphatic invasion (*p* = 0.003), vascular invasion (*p* = 0.025), and adjuvant chemotherapy (*p* = 0.024) were selected for multivariate analyses. The multivariate analyses showed that sex [male vs. female, hazard ratio (HR) 3.09, 95% confidence interval (CI) 1.58–6.48, *p* < 0.001], LNR (≥ 0.06 vs. < 0.06, HR 2.35, 95% CI 1.13–5.17, *p* = 0.021), tumor diameter (< 38 vs. ≥ 38 mm, HR 2.57, 95% CI 1.30–5.09, *p* = 0.007), and tumor location (right-sided vs. left-sided, HR 2.11, 95% CI 1.01–4.46, *p* = 0.047) were significant independent prognostic factors for the OS. Furthermore, for the RFS, the sex (male vs. female, HR 2.02, 95% CI 1.09–3.90, *p* = 0.026) and LNR (≥ 0.06 vs. < 0.06, HR 4.76, 95% CI 2.08–11.64, *p* < 0.001) were significant independent prognostic factors. Subgroup analyses for the OS and RFS were done for the sex (male vs. female) and LNR (≥ 0.06 vs. < 0.06). All patients were subdivided into 4 groups as follows: female with LNR < 0.06, male with LNR < 0.06, female with LNR ≥ 0.06, and male with LNR ≥ 0.06. In addition, all patients were subdivided into 2 groups as male with LNR ≥ 0.06 and others. The OS and RFS curves and rates are shown in Fig. 2 and Table 6, respectively. Female patients with LNR < 0.06 had a significantly better OS and RFS than male patients with LNR ≥ 0.06 (both *p* < 0.001). Furthermore, male patients with LNR ≥ 0.06 had a significantly worse OS and RFS than the others (both *p* < 0.001).

**Table 4:** Univariate prognostic analyses in pT4 colon and rectosigmoid cancer (*N* = 130)

Parameters	Categories	Number (%)	5-year OS (%)	*p* value	5-year RFS (%)	*p* value
Age (years)	≥ 74	30 (23.1)	59.0	**0.003**	63.3	0.791
	< 74	100 (76.9)	82.7		63.6	
Gender	Male	73 (56.2)	70.5	**0.003**	55.5	**0.033**
	Female	57 (43.8)	85.7		73.6	
Body mass index	≥ 21	82 (63.1)	77.7	0.103	60.4	0.388
	< 21	48 (36.9)	76.5		68.8	
ASA score^a^	1/2	118 (90.8)	78.3	0.178	64.0	0.597
	3	12 (9.2)	66.7		58.3	
CEA (ng/ml)	≥ 4.8	60 (46.2)	67.8	**0.011**	55.8	**0.070**
	< 4.8	70 (53.8)	85.5		69.9	
Tumor location	Right-sided	55 (42.3)	64.1	**0.068**	65.1	0.913
	Left-sided	75 (57.7)	86.5		62.5	
Anastomosis	DST	68 (52.3)	86.6	0.200	60.1	0.609
	FEEA	62 (47.7)	66.6		67.5	
cT	cT3	32 (24.6)	74.2	0.824	65.3	0.822
	cT4a	98 (75.4)	78.2		62.9	
cN	cN0/1	125 (96.2)	77.2	0.485	63.9	0.617
	cN2	5 (3.8)	80.0		50.0	
pT	pT4a	125 (96.2)	76.4	0.280^b^	62.0	0.128^b^
	pT4b	5 (3.8)	100.0		100.0	
pN	pN0/1	103 (79.2)	78.2	0.275	68.8	**0.045**
	pN2	27 (20.8)	73.7		41.7	
Harvested lymph nodes	≥ 17	79 (60.8)	81.9	0.169	64.1	0.871
	< 17	51 (39.2)	70.4		62.6	
Lymph node ratio^c^	≥ 0.06	67 (51.5)	69.6	< **0.001**	42.2	< **0.001**
	< 0.06	63 (48.5)	85.3		85.7	
Tumor differentiation	Well/moderately	120 (92.3)	78.8	0.135	65.7	**0.097**
	Poorly	10 (7.7)	56.3		34.3	
Lymphatic invasion	ly0/1/2	115 (88.5)	80.6	**0.009**	68.5	**0.003**
	ly3	15 (11.5)	50.6		21.8	
Vascular invasion	v0/1/2	97 (74.6)	83.2	**0.038**	68.6	**0.025**
	v3	33 (25.4)	59.4		48.5	
Tumor diameter (mm)	≥ 38	87 (66.9)	79.8	**0.047**	67.5	0.217
	< 38	43 (33.1)	71.9		55.4	
Adjuvant chemotherapy	Absence	63 (48.5)	78.9	0.602	74.4	**0.024**
	Presence	67 (51.5)	75.7		53.1	
Operative time (min)	≥ 230	40 (30.8)	79.1	0.506	61.4	0.855
	< 230	90 (69.2)	76.4		64.3	
Blood loss (ml)	≥ 35	39 (30.0)	81.6	0.401	71.3	0.318
	< 35	91 (70.0)	75.5		60.2	
Conversion to open surgey	Absence	125 (96.2)	77.2	0.782	62.2	0.156^b^
	Presence	5 (3.8)	80.0		100.0	
Morbidity (Clavien–Dindo^d^ ≥ Grade II)	Absence	117 (90.0)	77.3	0.456	62.0	0.292
	Presence	13 (10.0)	76.2		76.9	

*DST* double stapling technique, *FEEA* functional end-to-end anastomosis, *RFS* recurrence-free survival, *OS* overall survival

Bold values was defined reaching p values < 0.1 in all variables

^a^American Society of Anesthesiologists score

^b^Log-rank-test

^c^The ratio of metastatic lymph nodes to total number of harvested lymph nodes

^d^Clavien–Dindo classification

**Table 5:** Multivariate prognostic analyses in pT4 colon and rectosigmoid cancer (*N* = 130)

Prognostic factors	Hazard ratio	95% CI	*p* value
OS			
Age (≥ 74 against < 74) (years)	1.75	0.80–3.72	0.159
Gender (male against female)	**3.09**	1.58–6.48	< **0.001**
CEA (≥ 4.8 against < 4.8) (ng/ml)	1.64	0.87–3.17	0.128
Tumor location (right-sided against left sided)	**2.11**	1.01–4.46	**0.047**
Lymph node ratio^a^ (≥ 0.06 against < 0.06)	**2.35**	1.13–5.17	**0.021**
Lymphatic invasion (ly3 against ly0/1/2)	1.88	0.82–4.03	0.132
Vascular invasion (v3 against v0/1/2)	1.91	0.96–3.70	0.066
Tumor diameter (< 38 against ≥ 38) (mm)	**2.57**	1.30–5.09	**0.007**
RFS			
Gender (male against female)	**2.02**	1.09–3.90	**0.026**
CEA (≥ 4.8 against < 4.8) (ng/ml)	1.52	0.83–2.84	0.176
pN (pN0/1 against pN2)	1.32	0.66–2.71	0.429
Lymph node ratio^a^ (≥ 0.06 against < 0.06)	**4.76**	2.08–11.64	< **0.001**
Tumor differentiation (well/moderately against poorly)	1.20	0.40–4.05	0.754
Lymphatic invasion (ly3 against ly0/1/2)	2.18	0.86–5.00	0.096
Vascular invasion (v3 against v0/1/2)	1.49	0.75–2.83	0.248
Adjuvant chemotherapy (absence against presence)	1.05	0.52–2.06	0.880

*CI* confidence interval, *RFS* recurrence-free survival, *OS* overall survival

^a^The ratio of metastatic lymph nodes to total number of harvested lymph nodes

**Table 6:** Survival rates of patients with pT4 colon and rectosigmoid cancer in a subgroup analysis (*N* = 130)

Parameters	Number (%)	5-OS (%)	*p* value	5-RFS (%)	*p* value
Female/LNR < 0.06	28 (21.5)	88.7	< 0.001	85.7	< 0.001
Male/LNR < 0.06	35 (26.9)	82.4		85.7	
Female/LNR ≥ 0.06	29 (22.3)	82.8		61.7	
Male/LNR ≥ 0.06	38 (29.2)	59.6		27.0	
Others	92 (70.8)	84.5	< 0.001	78.2	< 0.001
Male/LNR ≥ 0.06	38 (29.2)	59.6		27.0	

*LNR* lymph node ratio, the ratio of metastatic lymph nodes to the total number of harvested lymph nodes, *RFS* recurrence-free survival, *OS* overall survival

## Discussion

The guidelines from the European Association of Endoscopic Surgery (EAES) recommend that preoperative T4 colorectal cancer be treated with open resection [30]. In general, pT4 colon cancer requires technically demanding surgical procedures, including en bloc resection of adjacent infiltrated organs or structures. Open multivisceral resection for pT4 colon cancer is known to have a high postoperative morbidity and a high risk of microscopically positive surgical margins [12, 13]. Furthermore, it is often difficult to distinguish between tumor-induced inflammatory adhesions and pathological tumor involvement for locally advanced colon cancer, especially by laparoscopy. Some authors have argued that pT4 tumor can raise the risk of conversion to open surgery. In the COLOR trial, the overall conversion rate was 17%, whereas 50% of patients with pT4 tumor required conversion to open surgery [6]. Regarding previous reports with only T4 colorectal cancer, the conversion rate ranged from 5.6 to 24.7% [14–22, 31, 32]. In the present study, conversion to open surgery was required in only 5 patients (3.8%) with pT4 colon cancer. The Laparoscopic Colorectal Surgery Study Group reported a significantly lower conversion rate in high-volume centers than in low-volume centers [33]. Another previous report showed that the factors influencing conversion were 2 adherent organs (*p* = 0.028) and clinical suspicion of direct invasion to adjacent organs (cT4b) (*p* = 0.076) [34]. In the present study, the low conversion rate was likely due to the extensive experience of the colorectal surgical team and the exclusion of patients diagnosed with cT4b tumor on preoperative imaging. Indeed, although this study focused on pT4 cancer, the median operative time of 205 min, blood loss of 10 ml, and postoperative hospital stay of 7.5 days were comparable to those of large randomized trials of colorectal cancer of all TNM stages [4, 8]. Furthermore, some authors have shown no marked differences in morbidity and mortality between laparoscopic procedures and open surgery for T4 colorectal cancer [16–21, 32]. Morbidity rates ranged from 7.7 to 30% in these reports. In the present study, 13 patients (10.0%) had postoperative complications classified as Clavien–Dindo II and III, and there were no deaths at 30 days postoperatively.

The mean number of harvested lymph nodes was 19.9 (± 9.3) in the present study, and more than 12 lymph nodes were harvested in 107 patients (82.3%), which is in keeping with the guidelines of the National Comprehensive Cancer Network 2009 [35]. In the present study, 1 patient (0.8%) had a positive radial resection margin. R0 resection is generally recognized as the most important factor for achieving the best results in terms of local recurrence and the survival in T4 colorectal cancer [12, 36]. In the COLOR trial, 1% of patients with pT3 (4/350) had R1 resection, whereas 20% of patients with pT4 (6/30) had R1 resection [6]. In addition, a study reported the positive margin status for pT4 colon cancer for laparoscopic procedures and open surgery using a large US national surgical database [31]; they showed that patients who underwent laparoscopic colorectal resection did not have a significantly higher positive margin rate than patients who underwent open surgery (laparoscopy 26.2% vs. open surgery 24.3%, OR 1.10, *p* = 0.54). The present R1 rate is extremely low compared with previous reports, probably due to the exclusion of patients diagnosed as definitely having T4b tumor on preoperative imaging and the extensive experience of the dedicated laparoscopic surgical teams. These results suggest that laparoscopic surgery for pT4 colon cancer is safe and feasible with respect to surgical outcomes, with favorable conversion, morbidity, and R0 resection rates.

According to the large randomized trials for colorectal cancer, the 5-year OS and disease-free survival (or RFS) rates of laparoscopic procedures were 55–91 and 57–79%, respectively [5, 7, 10]. Despite including only pT4 cancer cases, the 5-year survival rates were comparable in the present study. The overall recurrence rate was 36.2% (47/130). This higher rate of recurrence can be explained by the oncological biology of pT4 tumors, which have an increased tendency for lymph node and systemic spread [37]. The low chemotherapy rate of only 51.5% also adversely affected the survival in these patients. This low chemotherapy rate was due to an extremely low rate of induction of adjuvant chemotherapy in pStage II (12.8%, patients of 6/47). The peritoneal recurrence rate was 9.2% (12/130) in the present study. T4 tumor with adherence caused by inflammation requires en bloc resection. The easier dissection may lead to a detrimental effect on the dissemination of tumor cells [36]. There is a possibility that this higher rate of peritoneal recurrence is due to careless surgical procedures, and it can also be explained by the oncological biology of pT4 tumors.

A systematic review of laparoscopic surgery for T4 colon cancer was recently reported [32]. It included 13 observational cohort studies published between 2012 and 2017, consisting of a total of1217 patients who underwent laparoscopic procedures and 1357 who underwent open surgery. T4 staging included both pT4 and cT4. There were no significant differences between the rates of laparoscopic procedure and open surgery in oncological outcomes, including the radicality of resection, and any survival measures. Furthermore, the rate of postoperative complications was significantly lower after laparoscopic surgery than after open surgery (RR 0.65, *p* < 0.001). However, the authors cautiously concluded that laparoscopic procedures for T4a tumor might be safe, whereas they seem less appropriate for T4b tumors requiring multivisceral resection, because the current literature does not provide a definitive answer on the oncological safety of minimally invasive surgery for locally advanced colon cancer. Furthermore, according to a large randomized trial for colorectal cancer, JCOG 0404, patients with cT4 who underwent laparoscopic surgery tended to show a worse survival than those in the open surgery group (HR 1.27, 95% CI 0.68–2.34) [10]. These results suggest that patients with T4b on surgical findings should be excluded from the laparoscopic approach.

In previous reports, the prognostic factors of laparoscopic surgery for pT4 colorectal cancer were not determined. In the present study, the multivariate analyses showed that a male sex (HR 3.09, 95% CI 1.58–6.48, *p* < 0.001 for OS; HR 2.02, 95% CI 1.09–3.90, *p* = 0.026 for RFS), LNR ≥ 0.06 (HR 2.35, 95% CI 1.13–5.17, *p* = 0.021 for OS; HR 4.76, 95% CI 2.08–11.64, *p* < 0.001 for RFS), tumor maximum diameter < 38 (HR 2.57, 95% CI 1.30–5.09, *p* = 0.007 for OS), and right-sided colon cancer (HR 2.11, 95% CI 1.01–4.46, *p* = 0.047 for OS) were significant independent factors related to a poor prognosis. Furthermore, in subgroup analyses, male patients with LNR ≥ 0.06 had a significantly worse OS and RFS (both *p* < 0.001). For the vast majority of cancers, the age-adjusted mortality rates are higher among male patients than females [38]. Furthermore, the survival after colorectal cancer resection is longer in female than in male patients [39, 40]. In previous reports using the Surveillance, Epidemiology and End Results (SEER) database, although female patients presented more emergently and at an older age and received less aggressive medical treatment than males, which is associated with a poorer long-term survival, they had a longer survival duration than male patients with colorectal cancer (HR 0.80, *p* < 0.001) [40]. Anatomy is unlikely to explain the differences in the survival because there are few differences in the technical difficulty of colon resection between male and female patients. Some authors speculate that differences in the circulating hormones or in the immunologic response to tumor between male and female patients may have been responsible for the survival advantage in female patients [39, 41–43]. They argued that a poor survival rate in male patients may be the result of an ongoing inflammatory response in the form of elevated C-reactive protein levels. Indeed, elevated C-reactive protein levels are more detrimental in male patients than in female ones [39, 41]. Other authors speculate that circulating estrogen stimulates a protective immune response to a tumor, whereas circulating testosterone results in a detrimental immune response [42, 43].

LNR has shown prognostic significance in colorectal cancer [23–26]. The conventional staging system for patients with colorectal cancer is based on the AJCC TNM staging system [11]. This staging is based on the total number of positive nodes and the depth of tumor invasion and is used as a prognostic tool. Several studies have directly compared LNR to N staging of TNM and found LNR to be superior in the staging of colon cancer [23–25]. In a previous report, LNR was a valuable prognostic factor in node-positive colon cancer for laparoscopic surgery [26]. Those authors showed that patients with LNR < 0.13 had the same long-term outcomes as stage II node-negative patients. In the present study, despite a different LNR cut-off value using an ROC curve analysis, pStage III patients with LNR < 0.06 had similar long-term outcomes to pStage II patients (81.3 vs. 86.6%, *p* = 0.772 for the 5-year OS, data not shown). In the subgroup analyses, the group of male patients with LNR ≥ 0.06 tended to have a worse prognosis than male patients with pStage IIIC (59.6 vs. 73.7%, *p* = 0.152 for the 5-year OS, data not shown).

Whether or not the tumor size significantly predicts the prognosis in colorectal cancer remains controversial. Some authors have shown that the tumor size that reflected the horizontal growth, which was correlated with advanced stage, and was an independent prognostic factor in colon cancer on multivariate analyses [44, 45]. Generally, it is recognized that the survival decreases with increasing tumor size. However, some reports have shown that a small tumor size was an unfavorable prognostic factor, with a decreased survival, in colon cancer [45, 46]. They showed that the 8-year cause-specific survival (CSS) of patients with stage IIA colon cancer was significantly lower with tumor size ≤ 25 mm than with tumor size > 26 mm (*p* = 0.003) [45]. In addition, in reports using the SEER database, very small breast cancer with positive lymph node involvement had an increased cancer-specific mortality compared with large tumors [47]. As a potential explanation of the present findings, we hypothesized that a small tumor size (< 38 mm) may be a surrogate marker for biological aggressiveness, resulting in an inferior OS compared with larger tumors, and indicating that the initial biological heterogeneity of colon cancers determines a tumor’s distinct growth pattern and invasive and metastatic abilities (71.9 vs. 79.8%, *p* = 0.040 for the 5-year OS, data not shown). These results suggest that patients with such poor prognostic factors may need stronger postoperative or preoperative adjuvant chemotherapy.

In conclusion, while this study was limited by being a retrospective study performed at a single institution, its results suggest that laparoscopic surgery for locally advanced pT4 colon cancer is safe and feasible, does not increase the conversion rate, does not increase the postoperative morbidity, and has oncological outcomes that are acceptable. Although there is still room for improvement in patients with poor prognostic factors, based on the present findings and the availability of a colorectal surgical team with appropriate expertise, select patients with locally advanced colon cancer should not be excluded from laparoscopic surgery. The criteria for the selection of patients and the level of surgeon experience must be defined more precisely in future prospective studies.
